# Pitfalls in the diagnosis of common benign bone tumours in children

**DOI:** 10.1007/s13244-014-0356-y

**Published:** 2014-09-26

**Authors:** Dana I. Dumitriu, Renaud Menten, Philippe Clapuyt

**Affiliations:** Department of Radiology, Pediatric Radiology Unit, Cliniques Universitaires Saint Luc, Université Catholique de Louvain, Avenue Hippocrate no. 10, 1200 Brussels, Belgium

**Keywords:** Bone tumour, Paediatric, Benign, Pitfalls, Imaging

## Abstract

Benign bone tumours in children are frequent lesions, often with a typical and very identifiable radiological presentation. However, their natural evolution and complications may be the source of variations and errors in interpretation. It is therefore important to understand the possible sources of change in the radiological aspect and to be familiar with common pseudotumoral lesions. The main aim of this review is to review typical aspects of the most common benign bone tumours in children, as well as less frequent variants of these tumours.

*Teaching points*

• *Benign bone tumours in children may have atypical radiological presentations.*

• *Some normal variants are commonly misinterpreted as tumours.*

• *X-ray is the main imaging tool for focal bone lesions.*

• *Depending on the X-ray, complementary imaging examinations and biopsy may be necessary.*

## Introduction: clinical presentation and general diagnostic approach

Benign focal bone lesions are commonly incidental imaging findings in children. Alternatively, they may be the cause of pain, a palpable mass or a pathologic fracture [1]. In all of these instances, the radiologist’s involvement is crucial in the initial description, differential diagnosis and further imaging strategy, if necessary.

**Fig. 1:**
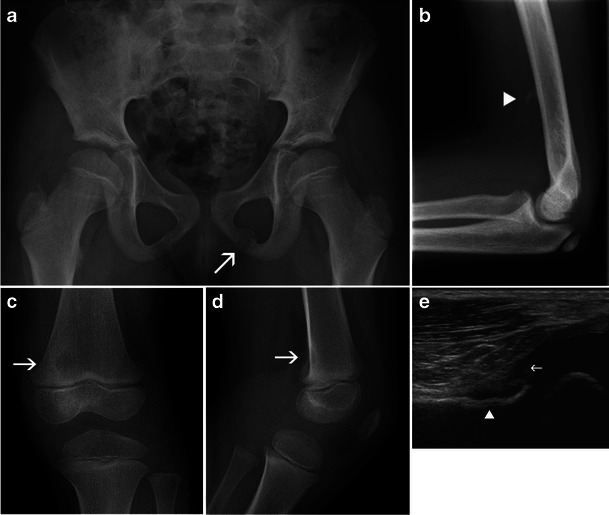
Normal variants commonly misinterpreted as bone tumours: **a** Asymmetric closure of the ischio-pubic synchondrosis, with a pseudotumoral aspect on the left side (*arrow*). **b** Supra-condylar process of the humerus (*arrowhead*). **c**, **d** Cortical desmoid of the distal femoral metaphysis (*arrows*). **e** Ultrasound: longitudinal scan of the posterior femoral metaphysis demonstrates the bone irregularity of a cortical desmoid (*arrowhead*) and the insertion of the medial gastrocnemius head (*arrow*)

When confronted with a focal bone lesion in a child, the radiologist must follow a logical algorithm, providing a systematic description of the lesion (age of the patient, location, type of bone, bone segment, limits, matrix, cortex, periosteal reaction, soft tissue mass) and the answers to the following questions:

- Could the lesion be a normal variant (Fig. 1a-e)?
- Is it a “leave me alone” (or “don’t touch”) lesion—a lesion of typical benign radiological aspect requiring no further imaging, treatment or specific follow-up [2] (Table 1)?
- Does the lesion have features suggestive of an aggressive nature (Table 2)?
- Is further imaging necessary and which technique is best adapted to the residual questions?
- Is a biopsy of the lesion required?

**Table 1:** Most common “leave me alone” lesions in the paediatric population [2]

Nonossifying fibroma/cortical defect/cortical desmoid
Fibrous dysplasia
Exostosis
Solitary bone island (osteoma)
Enchondroma

**Table 2:** Radiological features of nonaggressive versus aggressive/undetermined bone lesion

Feature	Nonaggressive	Aggressive/undetermined
Type of lysis	Geographic	Moth-eaten/permeative
Margins	Well defined	Ill defined
Zone of transition to normal bone	Narrow	Wide
Bone contour	Expansion from slow growth	Destruction
Periosteal reaction	Smooth, uninterrupted	Aggressive types (sunburst, hair-on-end, linear interrupted, Codman triangle)
Soft-tissue mass	Absent	Present

Conventional X-ray is still the most important imaging technique for bone lesions, as it demonstrates certain defining aspects, which may be difficult to identify as such by other techniques. Therefore, it should always be part of the imaging algorithm of a focal bone lesion, even if the initial diagnosis was made by a more complex technique, such as CT or MRI.

Imaging techniques complementary to X-ray are most often not necessary, but when performed, must provide the answer to a specific question. A combination of CT, MRI and bone scan may be required, depending on the conventional X-ray presentation of the lesion. Thus, each case should be judged individually, keeping in mind the following general guidelines [1, 3]:

- Whenever a finer, more detailed characterisation of the cortical changes, bone matrix alteration and periosteal reaction is necessary, CT is the adequate technique. It offers a more detailed “radiological” view of the lesion and completes the findings of the X-ray related to bone changes.
- MRI is more appropriate to identify the presence of bone oedema as well as identify and quantify infiltration, extraosseous masses and soft tissue changes. As such it is essential in the characterisation of malignant lesions. However, an important caveat must be kept in mind: some very typical radiological lesions, such as nonossifying fibroma, may have nonspecific and sometimes challenging aspects on MRI. Thus, MRI is not adequate as an initial imaging technique, and problematic focal bone lesions incidentally found on MRI require a complementary X-ray.
- Even though it lacks specificity, a bone scan is especially useful to demonstrate whether a lesion is uni- or multifocal, an important element in the differential diagnosis. It may also have more specific applications, such as identifying the nidus of an osteoid osteoma in doubtful cases.

## Most common benign tumours in children

Table 3 lists the most frequent locations of benign bone tumours, keeping in mind that exceptions to these rules may occur in everyday practice.

**Table 3:** Most common locations of benign bone tumours

Tumour	Most common locations	Location in long bones
FCD/NOF	Around the knee joint (distal femur/proximal tibia)	Metaphysis
Exostosis (osteochondroma)	Long bones, pelvis, ribs	Metaphysis
Enchondroma	Phalanges hands/feet	Metaphysis, diaphysis
Chondroblastoma	Around the knee joint (distal femur/proximal tibia), proximal humerus	Epiphysis
Simple bone cyst	Proximal humerus, proximal femur	Metaphysis
Aneurysmal bone cyst	Long bones, pelvis, posterior elements of vertebrae	Metaphysis
Osteoid osteoma	Neck of femur, other long bones (especially lower limbs)	Diaphysis, metaphysis

### Fibrous cortical defect (FCD) and nonossifying fibroma (NOF)

FCD and NOF are both developmental fibro-osseous lesions with similar histological structures. The difference between the two entities is the size, with lesions under 2 cm being considered FCDs and lesions over 2 cm NOFs [1].

**Fig. 2:**
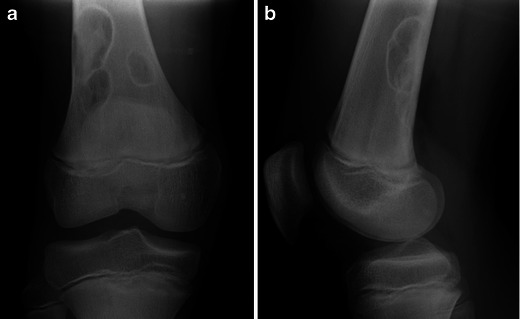
Frontal (**a**) and lateral (**b**) X-rays of the right knee: Non-ossifying fibroma—typical radiological aspect

Typically, they are intracortical well-defined lytic lesions, located in the metaphysis of long bones, most often around the knee joint (Fig. 2a and b).

If large, they may take a more “bubbly” aspect and extend toward the medullary bone. Even though they are not expansile lesions, the outer cortex may be thinned or slightly bulged.

**Fig. 3:**
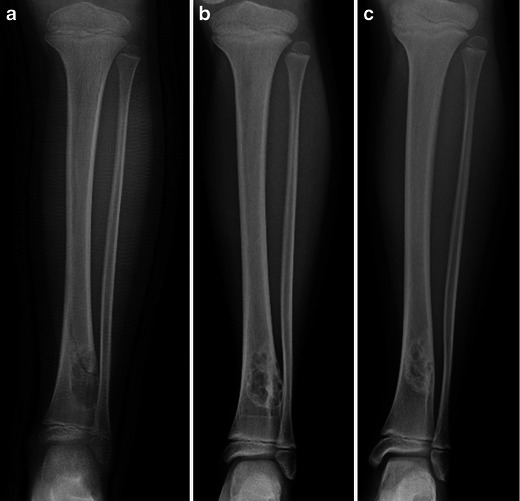
Frontal X-rays of the left tibia: Non-ossifying fibroma of the distal tibial metaphysis—evolution after fracture [age 7 (**a**), age 9 (**b**), age 11 (**c**)]

They may also be complicated by fractures, especially when large. The healing process, as well as the natural evolution of a NOF, causes changes in its aspect, rendering the correct diagnosis less straightforward. As such, in time they become increasingly sclerotic and move away from the physis with progressive bone growth (Fig. 3a–c).

**Fig. 4:**
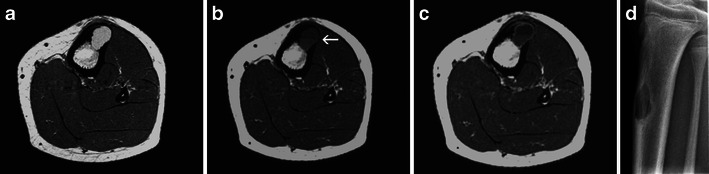
Non-ossifying fibroma—incidental finding on MRI: The lesion is hyperintense on T2 (**a**), hypointense on T1 with a fluid-fluid level (*arrow*; **b**), and it presents peripheral contrast enhancement (**c**). Lateral X-ray of the tibia demonstrates the typical NOF aspect (**d**)

An important pitfall in the diagnosis of FCDs and NOFs is their MRI aspect. Depending on their size, content and various phases of evolution, they may have variable signal intensities (Fig. 4a–c). Most commonly they are hypointense on both T1- and T2-weighted sequences. However, when still in the growth phase, they present T2 hyperintensity [4]. Contrast enhancement is common [4] and should not be a cause for misinterpretation.

NOFs may contain fluid-fluid levels (Fig. 4b), which are by no means specific to any given bone lesion, but merely demonstrate the occurrence of internal bleeding [5, 6]. An X-ray is imperative in order to clarify any doubt in relation to the MRI aspect of an FCD/NOF (Fig. 4d).

A lesion similar to FCD is the cortical desmoid of the distal femoral metaphysis. On a frontal X-ray of the knee, it may be the cause of confusion by appearing as a round, well-defined lucency (Fig. 1c and d). The origin of the lesion is probably repeated microavulsions at the insertion site of the adductor magnus and the medial gastrocnemius head [7, 8].

The diagnosis of a cortical desmoid relies on the typical location and the confirmation of its cortical origin on a lateral X-ray. In doubtful cases, ultrasound is useful to demonstrate the muscular insertion on the cortical defect area and the lack of a tissue mass (Fig. 1e). Even though the same information can be gathered from CT or MRI, these more sophisticated techniques are not necessary [3].

### Exostosis (ostechondroma)

Considered developmental defects of the growing bone or benign bone tumours of cartilaginous origin, exostoses are the most common benign bone lesion and may take various radiological aspects [1]. Classically they are divided into pedunculated (with a long stalk) and sessile (with a flat base) and their origin is the metaphyseal region of long bones. They may however originate from the pelvic bones or the ribs and take more bizarre, irregular aspects in these regions. Axial skeleton exostoses may be solitary, but they are more common in patients with hereditary osteochondromatosis (multiple exostoses; diaphyseal aclasis).

**Fig. 5:**
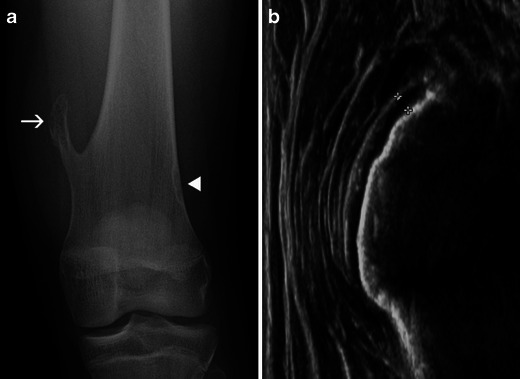
Pedunculated exostosis: **a** Frontal X-ray of the left knee demonstrates the typical features of exostosis (*arrow*); also note the cortical defect on the lateral femoral metaphysis (*arrowhead*). **b** Ultrasound measurement of the hypointense cartilage cap (*between calipers*)

The key to their correct diagnosis is demonstrating the continuity with the originating bone cortex, the direction of growth for pedunculated exostosis—parallel to the bone shaft, moving away from the originating metaphysis—and the presence of a cartilage cap. (Fig. 5a) This cartilage cap is demonstrated by imaging techniques with good soft tissue rendering, such as ultrasound or MRI. Given the very low rate of malignant transformation of exostoses [1] and the excellent spatial resolution, ultrasound is a perfect tool for the measurement of the cartilage cap, which should not exceed 1 cm in thickness (Fig. 5b).

**Fig. 6:**
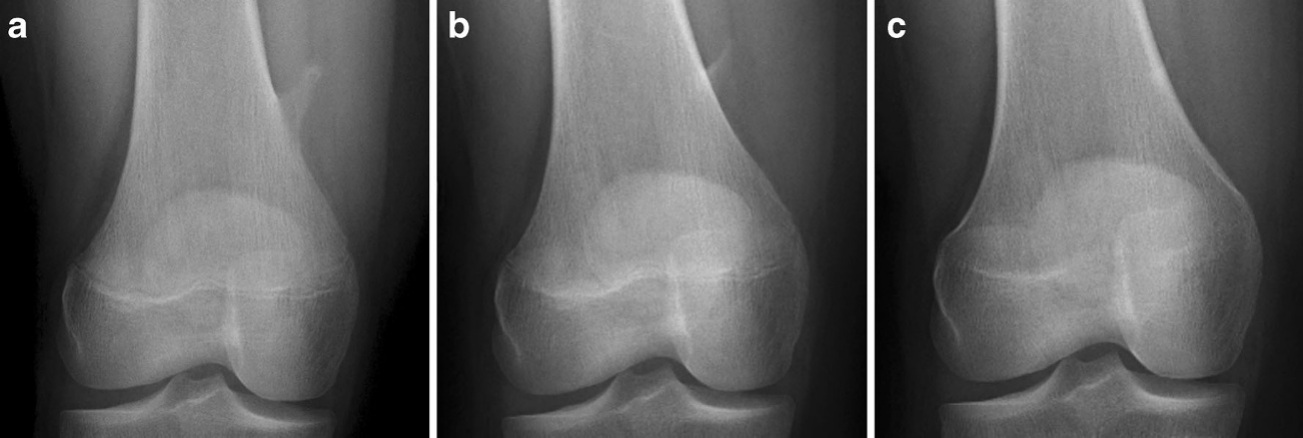
Exostosis of the distal femoral metaphysis—involution over 3 years [age 12 (**a**), age 13 (**b**), age 15 (**c**)]

Exostoses may compress adjacent structures; they may fracture or cause pain. In rare cases, especially after trauma with complete or incomplete fracture, they may disappear (Fig. 6a–c). In patients with hereditary osteochondromatosis, they may cause more significant deformities and interfere with normal metaphyseal growth.

One normal variant sometimes misinterpreted as an exostosis is the supracondylar process (Fig. 1b). This vestigial bone formation located on the anterior humeral diaphysis has an oblique direction and its tip is oriented toward the distal humeral metaphysis. It is connected to the medial epicondyle by the ligament of Struthers and may rarely be the cause of an entrapment syndrome of the median nerve [7]. Although it is also clearly identified by other imaging techniques, they are rarely necessary, as its location and radiological aspect are so typical. No follow-up or further imaging is required for this entity.

### Enchondroma

**Fig. 7:**
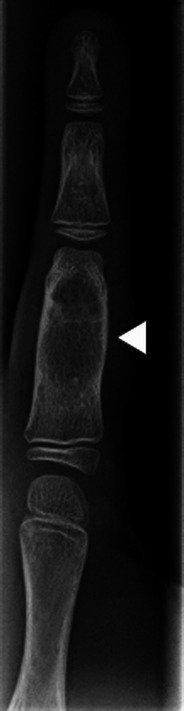
Enchondroma of the proximal phalanx (*arrowhead*). Note the small points of calcification at the proximal aspect of the lesion, highly suggestive of a cartilaginous matrix

Enchondromas are benign lesions of cartilaginous origin, appearing in most cases in the tubular bones of the hands and feet [1] (Fig. 7). They are of central location, but may grow asymmetrically and produce endosteal scalloping. The lesions contain a cartilaginous matrix, which is better characterised with CT, and share the same signal with cartilage on all MRI sequences.

**Fig. 8:**
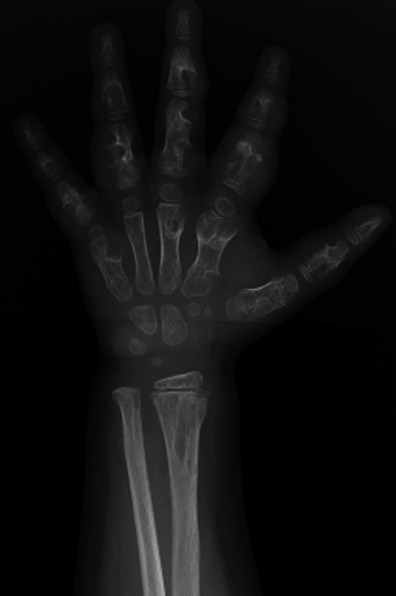
Ollier disease—multiple enchondromas of the hand

Bizarre and sometimes pseudo-aggressive aspects of enchondromas may be identified in multiple enchondromatosis syndromes, such as Ollier and Maffucci disease (Fig. 8).

**Fig. 9:**
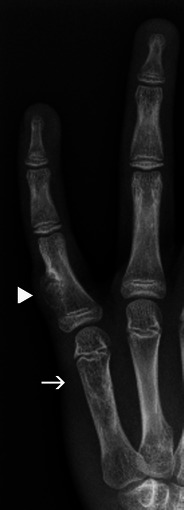
Enchondroma of the fifth metacarpal (*arrow*) and enchondroma protuberans (*arrowhead*) of the proximal phalanx

Another possible difficulty arises when the location of the enchondroma is not in the central expected position. Such variants are termed enchondroma protuberans and may occur in either the typical phalangeal location or the proximal humerus (Fig. 9). This variant may sometimes appear very exofitic and require better characterisation with CT or MRI to identify the cartilaginous matrix and lack of aggressive signs [9].

### Chondroblastoma

**Fig. 10:**
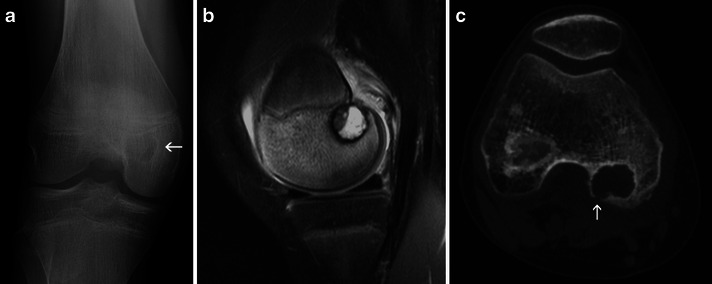
Typical chondroblastoma of the distal femoral epiphysis: **a** Frontal X-ray of the right knee: well-defined round osteolytic lesion of the distal femoral epiphysis (*arrow*). **b** Sagittal fat-saturated proton density MRI: the lesion is well defined, mostly hyperintense, surrounded by bone oedema and an inflammatory reaction of the adjoining soft tissues. **c** CT of the right knee: the cortex is interrupted on the posterior border of the lesion (*arrow*), but no periosteal reaction is identified

Although it is one of the few epiphyseal focal bone lesions, the diagnosis of chondroblastoma is not always straightforward. Typically it is a well-defined lytic lesion, with a cartilaginous matrix, limited to the epiphysis, appearing in the second decade of life [1] (Fig. 10a–c).

**Fig. 11:**
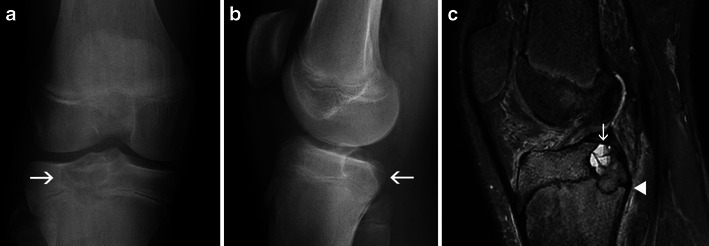
Chondroblastoma of the proximal tibial epiphysis: Frontal (**a**) and lateral (**b**) X-rays of the right knee demonstrate an osteolytic epiphyseal lesion, crossing the open physis and extending into the metaphysis. Sagittal fat-saturated proton density-weighted MRI (**c**) confirms the lesion and the extension across the physis (*arrowhead*). Note the presence of fluid-fluid levels (*arrow*)

Potential pitfalls in the diagnosis of this tumour arise from its evolution: it may extend into the metaphysis, especially if it develops after physeal closure (Fig. 11a and b). It may be associated with cortical interruption and periosteal reaction, which should not be interpreted as signs of malignancy in the absence of a soft tissue mass. It is also not uncommon for fluid-fluid levels to be identified in a chondroblastoma [5], making the differential diagnosis from an aneurysmal bone cyst more challenging, especially if the lesion is large (Fig. 11c).

One helpful element is the very intense inflammatory response this tumour causes, which is adequately identified by MRI (Fig. 10b)—in the absence of signs of infection, which orient the diagnosis toward osteomyelitis, this is a helpful sign to distinguish chondroblastoma from other entities [1, 3].

Although extremely rare, metaphyseal or even diaphyseal locations have been reported for chondroblastoma [10, 11]. These cases always require histologic confirmation.

### Simple bone cyst

Simple bone cysts are well-defined lucent cystic lesions, centred in the bone, usually located in the proximal humerus or femur. Although termed unicameral bone cysts, these benign lesions do in fact often contain some septa. This is mainly related to the fact that the most common presentation of the cyst is by pathological fracture. Otherwise, they may be incidental findings. The healing process of the fracture induces changes in the simple, unique cystic aspect, with periosteal thickening, septa and contrast enhancement of the cyst lining on MRI. All of these should not be interpreted as signs of aggressiveness.

**Fig. 12:**
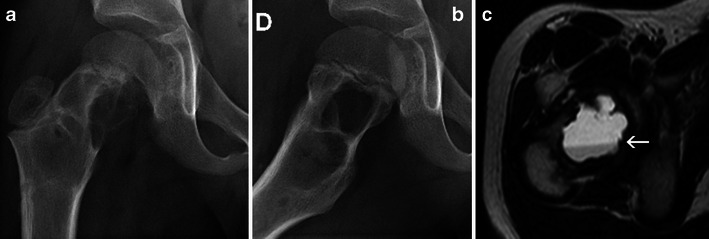
Simple bone cyst of the right femoral neck: Frontal (**a**) and lateral (**b**) X-rays of the right femur: well-defined central lytic lesion, with internal septa. Transverse T2-weighted MRI (**c**): a unique, large fluid-fluid level is identified (*arrow*)

Bleeding may occur within the cystic cavity and fluid-fluid levels are not uncommon (Fig. 12a–c).

In time, the physis moves away from the cyst, which becomes diaphyseal. It may also become more sclerotic, with or without treatment.

### Aneurysmal bone cyst (ABC)

One of the trickier benign bone tumours is the aneurysmal bone cyst, which is considered more of a post-traumatic accumulation of blood and fluid than a tumoral proliferation in itself. It may be isolated or an associated component of another benign or malignant tumour [12].

**Fig. 13:**
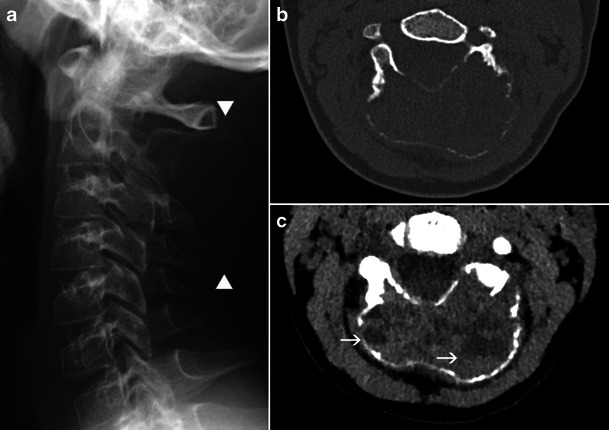
Aneurysmal bone cyst of the second cervical vertebra (C2): **a** Lateral X-ray of the cervical spine: large expansile lytic lesion of the posterior arch C2 (*between arrowheads*). **b** CT, axial slice, bone windowing: confirmation of the lesion, with extreme thinning and focal interruption of the bone cortex. **c** CT, axial slice, soft-tissue windowing: fluid-fluid levels (*arrows*) are identified inside the cyst

**Fig. 14:**
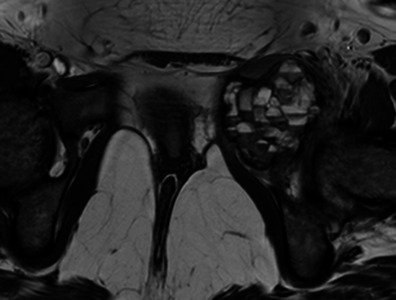
Transverse T2-weighted MRI: aneurysmal bone cyst of the left pubic branch, with multiple small fluid-fluid levels

Typically, it is an eccentric expansile multiloculated lesion, often located in the metaphysis of a long bone, sometimes the pelvis or the posterior elements of vertebrae (Fig. 13a and b). Often considered the most typical aspect of the lesions, multiple fluid-fluid levels in small size loculations are identified by CT (Fig. 13c) or MRI (Fig. 14). With the very expansile nature of the lesion sometimes come cortical disruption and periosteal reaction, which are not necessarily a cause for concern [12].

No matter how typical the position and aspect of the lesion, histological confirmation is always necessary, especially to differentiate ABCs from the rare telangiectatic osteosarcoma, which is very similar in macroscopic appearance [13]. Fluid-fluid levels should not be considered more than a suggestive element, as they may appear in an array of benign or malignant lesions, as soon as internal bleeding occurs [5, 6].

### Osteoid osteoma

Osteoid osteoma is one of the most identifiable benign bone tumours when its clinical and radiological presentations are typical. It is classically a cortical lesion, located in the diaphysis or metaphysis of long bones, the femoral neck being the most common location [1]. However it may be subperiosteal, endosteal or medullary, with most authors considering these locations to result from migration from the typical cortical origin [14, 15].

**Fig. 15:**
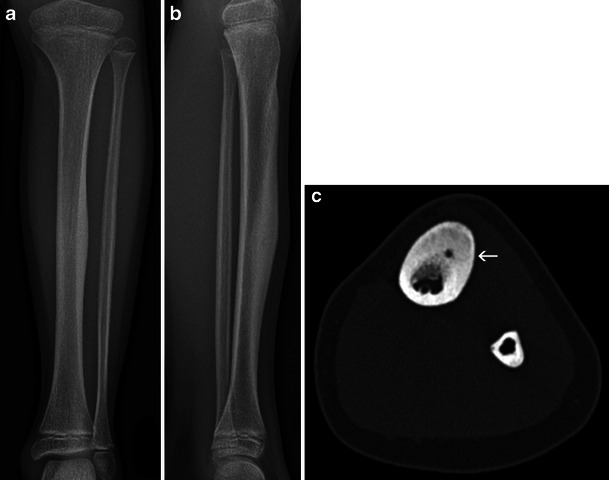
Osteoid osteoma of the antero-lateral tibial shaft: Frontal (**a**) and lateral (**b**) X-rays of the left tibia: osteosclerotic thickening of the antero-medial diaphyseal cortex. **c** Axial CT slice of the tibial shaft identifies a small lucent nidus (*arrow*) within the intense sclerotic cortical proliferation

The tumour is represented by a lucent nidus of vascular osteoid tissue, containing a dense sequestrum-like fragment. The absence of this dense fragment does not exclude the diagnosis (Fig. 15a–c). The central nidus, which may vary in size from a few mm to 1.5 cm, is surrounded by reactive periosteal new bone formation, which may sometimes be very dense and hide the nidus.

Although MRI and bone scans are useful to detect an anomaly, the inflammatory changes may sometimes be too diffuse and a small nidus overlooked. The most adequate technique to correctly identify the nidus is CT (Fig. 15c), which is also used subsequently to guide radiofrequency ablation therapy [15].

**Fig. 16:**
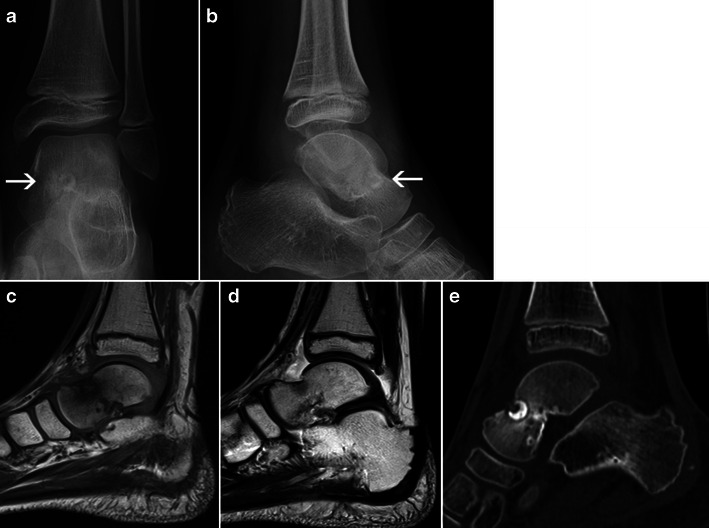
Osteoid osteoma of the left talar neck: Frontal (**a**) and lateral (**b**) X-rays of the left ankle: large round sclerotic fragment within an osteolytic cavity (*arrows*); no surrounding reactive osteosclerosis is identified. **c** Sagittal T1-weighted MRI: intense bone oedema surrounding the lesion. **d** Sagittal T2-weighted MRI: an intense inflammatory reaction is identified within the ankle joint. **e** CT, sagittal reconstruction: confirmation of the large nidus, entirely filled by the sclerotic fragment and with almost no surrounding osteosclerosis

Despite the clear-cut imaging aspects in typical cases, the diagnosis of osteoid osteoma can be very challenging whenever the dense sclerotic reaction is absent or minimal. This occurs especially in the case of intra- or juxta-articular osteoid osteomas (which are often superiosteal or intramedullary) [16]. Because of the proximity to the joint, the inflammatory reaction can be so rich and the production of bone so slight that the nidus is hard to identify (Fig. 16a–e). Unexplained persistent inflammatory arthritis, resistant to treatment, should prompt the clinician and the radiologist to consider the possibility of an occult osteoid osteoma [16].

## Conclusion

The radiological diagnosis of benign bone tumours in children is not always as simple as it may seem. Understanding which events and processes induce variations of the classical imaging presentations and knowing to distinguish normal variants and identify signs of an aggressive process are the key to a correct diagnostic algorithm. It is the radiologist’s responsibility to indicate whether complementary imaging techniques are necessary, which technique is appropriate for the question at hand and whether a biopsy of the lesion is required.
